# Recent Synthetic Applications of the Hypervalent Iodine(III) Reagents in Visible-Light-Induced Photoredox Catalysis

**DOI:** 10.3389/fchem.2020.551159

**Published:** 2020-09-23

**Authors:** Chaoyue Chen, Xin Wang, Tinghai Yang

**Affiliations:** ^1^School of Chemistry and Environmental Engineering, Jiangsu University of Technology, Changzhou, China; ^2^State Key Laboratory of Coordination Chemistry, School of Chemistry and Chemical Engineering, Nanjing University, Nanjing, China

**Keywords:** hypervalent iodine reagent, photoredox catalysis, photochemistry, radical intermediate, synthetic methods

## Abstract

The synergistic combination of visible-light-induced photoredox catalysis with hypervalent iodine(III) reagents (HIRs) represents a particularly important achievement in the field of hypervalent iodine chemistry, and numerous notable organic transformations were achieved in a mild and environmentally benign fashion. This account intends to summarize recent synthetic applications of HIRs in visible-light-induced photoredox catalysis, and they are organized in terms of the photochemical roles of HIRs played in reactions.

## Introduction

During the past several decades, the chemistry of hypervalent iodine reagents (HIRs) has gained more and more attention due to their unique electrophilic properties (Brand et al., 2011; Charpentier et al., 2015), valuable oxidizing abilities (Yoshimura and Zhdankin, 2016; Wang and Studer, 2017), and environment friendly features (Zhdankin, 2013; Yoshimura and Zhdankin, 2016). The special structural features and unparalleled reactivities of HIRs lie in their unique 3-center-4-electron (3c-4e) bonds (L—I(III) —X), which are highly polarized and are longer and weaker than classical covalent bonds (Zhdankin, 2013; Yoshimura and Zhdankin, 2016; Jia and Chen, 2018). Generally, HIRs offer multiple advantages for synthetic organic chemistry: (i) mild and highly chemoselective oxidizing properties; (ii) benign environmental character; (iii) commercial availability; and (iiii) convenient structural modification (Brand et al., 2011; Zhdankin, 2013; Li Y. et al., 2016; Yoshimura and Zhdankin, 2016; Hari et al., 2018). These advantages of HIRs give synthetic chemists the opportunities to design and access novel and more challenging reactions. As a result, a wide array of organic transformations ranging from oxidative coupling processes (Wang and Liu, 2016; Jia and Chen, 2018), ligand transfer reactions (Zhdankin, 2013; Yoshimura and Zhdankin, 2016), rearrangements (Zhdankin, 2009; Brand et al., 2011), C–C, C–O or C–N bond formations (Li Y. et al., 2016; Hyatt et al., 2019) to numerous other reactions have recently been developed based on HIRs.

Since 2008, visible-light-induced photoredox catalysis has emerged as one of the most rapidly expanding fields in organic chemistry (Xuan and Xiao, 2012; Koike and Akita, 2014; Romero and Nicewicz, 2016; Shaw et al., 2016; Staveness et al., 2016; Twilton et al., 2017). In photoredox-catalyzed procedures, metal photocatalysts (iridium-, ruthenium-, and copper-based) or organic dyes (rose bengal, eosin Y, BODIPY, 4CzIPN, coumarins, and rhodamine derivatives) can efficiently convert visible light into chemical energy, thereby allowing the activation of organic substrates *via* single-electron transfer (SET) events, and eventually accessing to a large number of synthetically important reactions under very mild reaction conditions.

**Figure 1:**
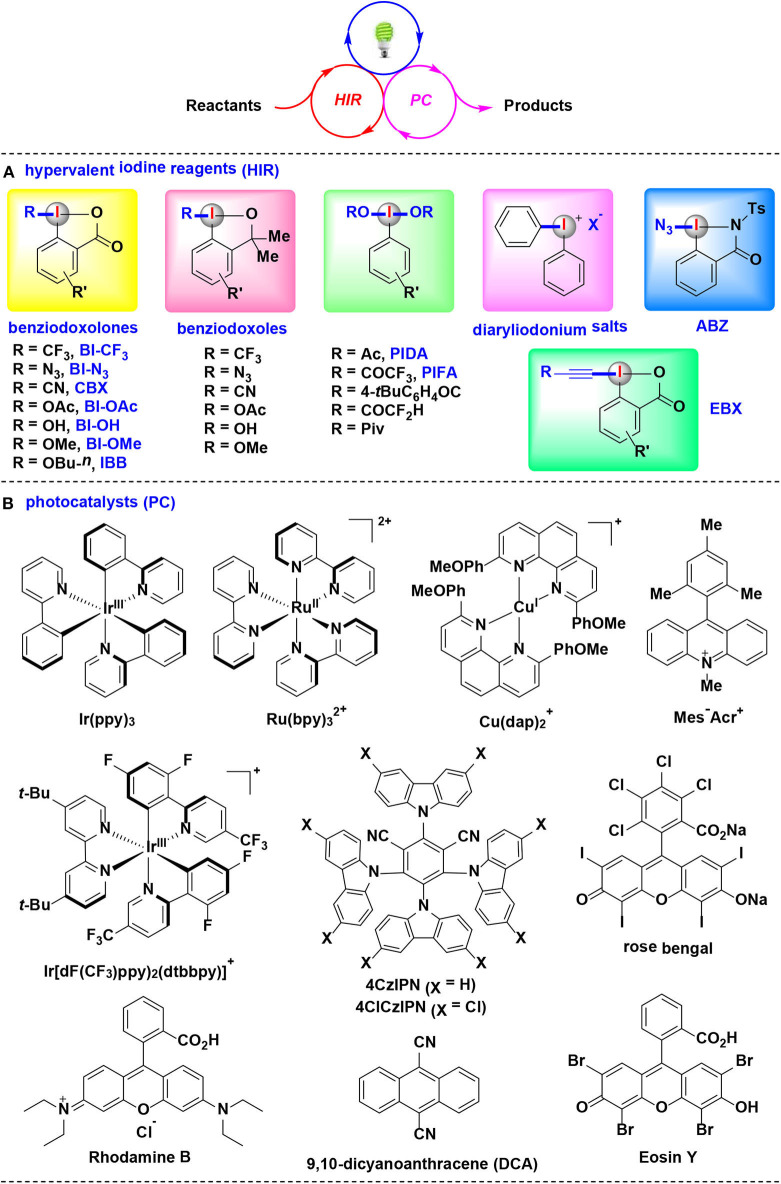
The synergistic combination of visible-light-induced photoredox catalysis with HIRs, and typical photocatalysts and HIRs using in this methodology.

Very recently, HIRs have quickly established themselves as efficient and versatile reaction partners for visible-light-induced photoredox catalysis. Many studies related to the elegant merging of photoredox catalysis with HIRs have resulted in significant advancements (Wang and Liu, 2016; Wang and Studer, 2017; Jia and Chen, 2018). By the appropriate choice of HIRs, photocatalysts, light sources and solvents, a wide array of bond-forming reactions were developed in mild and environmentally benign fashion (Figure 1).

**Figure 2:**
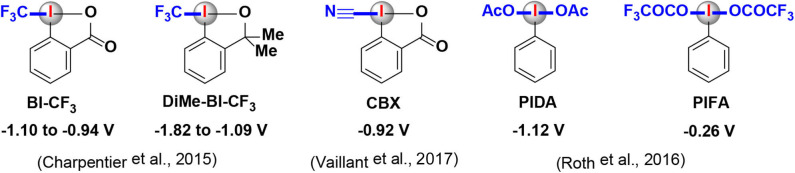
Redox potentials of HIRs that featured in this review.

Mechanistically, a typical photoredox catalytic cycle consists of a sequence of three key steps: a photoexcitation process followed by two SET processes. On account of the smooth occurrence of the SET processes, the redox (oxidation/reduction) potentials of both photocatalysts and HIRs must be taken into consideration in order to find the best-matched partners in a photoredox catalysis/HIR reaction. The oxidative/reductive abilities of commonly used transition metal and organic photocatalysts are relatively well investigated (Table 1) (Reckenthaler and Griesbeck, 2013; Koike and Akita, 2014; Romero and Nicewicz, 2016; Roth et al., 2016; Lemos et al., 2019). However, despite the practical significance of HIRs, redox potentials of them has not been sufficiently evaluated until now, only limited of redox potential values of HIRs were reported in literatures (Figure 2) (Charpentier et al., 2015; Roth et al., 2016; Vaillant and Waser, 2017). Just in 2020, Radzhabov and coworkers reported new calculated values of the relative redox potentials of [bis(acetoxy)iodo]-arenes (Radzhabov et al., 2020). The influence of various substituents and the effects of various solvents on the reduction potentials of HIRs was both detailed evaluated. This theoretical assessments may provide a useful reference for the design of new photoredox reactions based on ArI(OAc)_2_.

**Table 1:** Redox potentials of typical photocatalysts that featured in this review.

**PC**	E_1/2_ (PC^+^/PC*)	E_1/2_ (PC*/PC^−^)	**E_1/2_ (PC^+^/PC)**	**E_1/2_ (PC/PC^−^)**	**References**
*fac*-*Ir*(*ppy*)_3_	−1.73	+0.31	+0.77	−2.19	(Lemos et al., 2019)
[Ru(bpy)_3_]^2+^	−0.81	+0.77	+1.29	−1.33	(Lemos et al., 2019)
[Ir(dF(CF_3_)ppy)_2_(dtbbpy)]^+^	−0.89	+1.21	+1.69	−1.37	(Lemos et al., 2019)
4CzIPN	−1.04	+1.35	+1.52	−1.21	(Lemos et al., 2019)
Eosin Y	−1.60	+1.18	+0.72	−1.14	(Reckenthaler and Griesbeck, 2013)
Rose bengal	−0.68	+0.99	+1.09	−0.78	(Reckenthaler and Griesbeck, 2013)
DCA	−1.01	+2.07	+1.89	−0.83	(Reckenthaler and Griesbeck, 2013)

*All potentials are given in volts in CH_3_CN vs. the saturated calomel electrode (SCE)*.

In line with photoredox catalysis, HIRs play two different kind of photochemical roles such as reagent for functional-group transfer and mild oxidant for substrates activation (Wang and Liu, 2016; Wang and Studer, 2017; Jia and Chen, 2018). HIRs bearing trifluoromethyl, azido, alkynyl, and cyano groups can readily participate in photocatalytic reactions for the transformation of perfluoroalkylation (Koike and Akita, 2016), azidation (Fumagalli et al., 2015), alkynylation (Kaschel and Werz, 2015), and cyanation (Le Vaillant et al., 2017), respectively. In contrast, hydroxyl-, alkoxyl-, and acetoxy- benziodoxoles (BI-OH, BI-OR, and BI-OAc) are usually acted as the oxidant for activation of carboxylic acids (Huang et al., 2016), alcohols (Liu et al., 2018) or alkyl C-H bonds (Li et al., 2017) for the generation of oxygen- or carbon-centered radicals under photoredox catalysis. In certain cases (Jia et al., 2016, 2018), two HIRs were employed in the same photoredox procedure: one of which acts as a reagent and the other serves as mild oxidant.

The review herein intends to summarize recent synthetic applications of HIRs in visible-light-induced photoredox catalysis. The document is organized in terms of the photochemical roles of HIRs played in reactions, with particular emphasis placed on the literature from 2016 until the end of March of 2020. In every section, we arrange the synthetic methods according to their reaction types.

## HIRs Act as Functional Group Transfer Reagents

### Fluoroalkylation

Visible-light photoredox catalytic methods have been proven to be one of the most efficient pathways for the incorporation of a variety of fluoroalkyl groups into organic skeletons (Koike and Akita, 2016). Both cyclic and acyclic HIRs possessing various fluorinated groups can serve as effective fluoroalkyl-transfer reagents in photoredox-catalyzed fluoroalkylation (Li Y. et al., 2016; Wang and Liu, 2016). In these processes, HIRs usually choose the oxidative quenching pathway to furnish the key fluoroalkyl radicals, thus enabling the synthesis of a wide variety of fluoroalkylated compounds.

**Figure 3:**
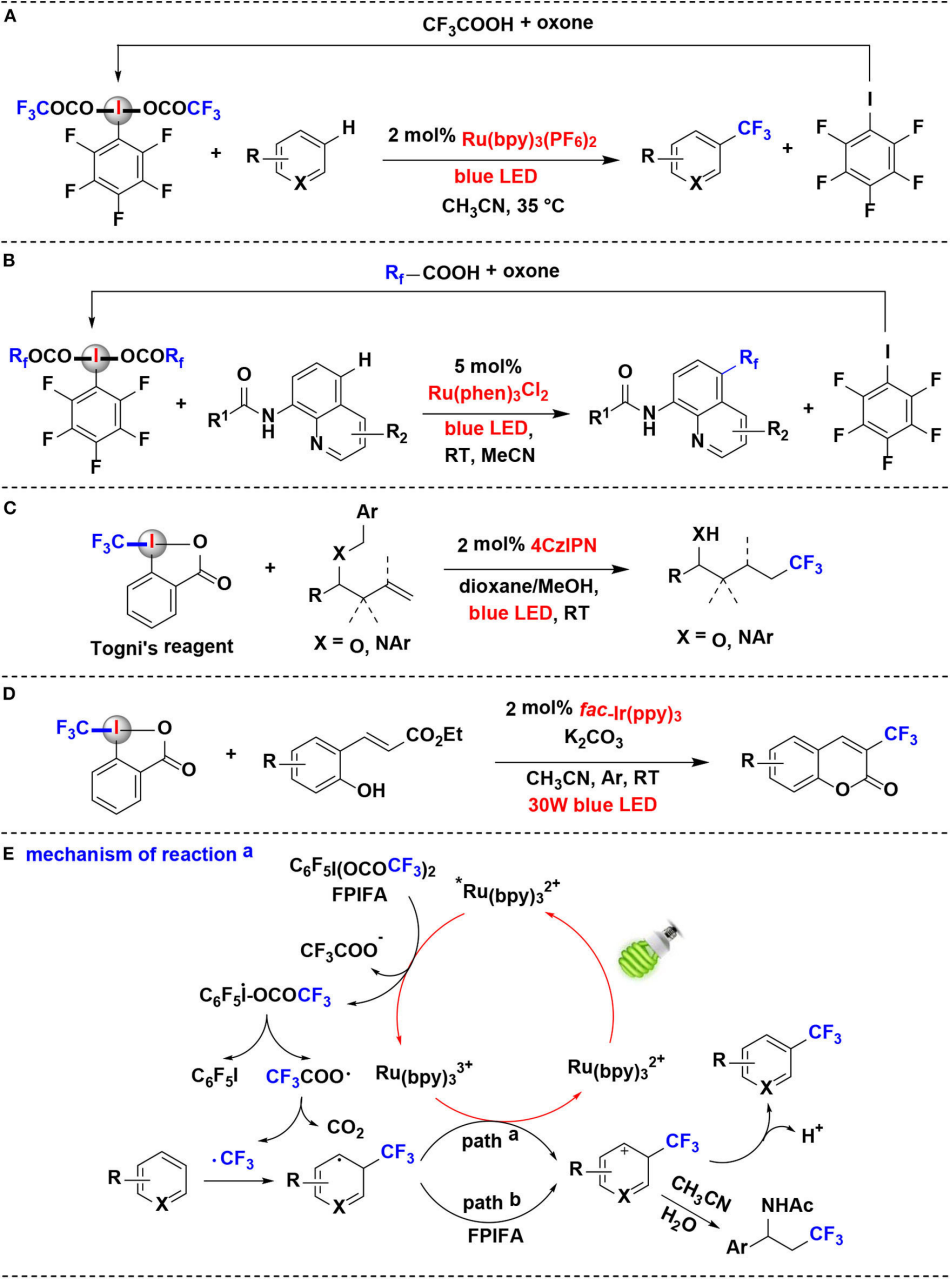
Photoredox-catalyzed fluoroalkylation using HIRs as fluoroalkyl-transfer reagents. **(A)** Trifluoromethylation of (hetero)arenes. **(B)** Perfluoroalkylation of aminoquinolines. **(C)** hydrotrifluoromethylation of benzyl-protected homoallylic alcohol and amine derivatives. **(D)** Trifluoromethylation of ortho-hydroxycinnamic esters. **(E)** Mechanism of reaction **(A)**.

In 2018, Qing and coworkers reported the decarboxylative trifluoromethylation of (hetero)arenes using ArI(OCOCF_3_)_2_ as CF_3_ source by ruthenium photoredox catalysis (Yang et al., 2018) (Figure 3A). A series of fluorinated ArI(OCOCF_3_)_2_ were examined and C_6_F_5_I(OCOCF_3_)_2_ (FPIFA) was proved to be the best option. Notably, FPIFA is easily accessible from C_6_F_5_I and TFA in the presence of oxone (Harayama et al., 2006; Zagulyaeva et al., 2010), and C_6_F_5_I could be recycled from the decarboxylation reaction in high yield.

The authors proposed the reaction mechanism depicted in Figure 3E. Initially, Ru(bpy)${}_{3}^{2+}$ is excited by visible light to generate the excited specie ^*^Ru(bpy)${}_{3}^{2+}$, which performs the SET process with FPIFA to afford the iodanyl radical, accompanied by the formation of Ru(bpy)${}_{3}^{3+}$. Then, the resulting iodanyl radical extrudes C_6_F_5_I to release the trifluoroacetoxy radical, which can undergo further scission, leading to the formation of CF_3_ radical. The CF_3_ radical thus attack the aromatic ring in arene to give aromatic radical. The aromatic radical might be oxidized either by Ru(bpy)${}_{3}^{3+}$ (path a) or by FPIFA (path b) to yield the corresponding aromatic cation. At last, the aromatic cation is converted into the target product through the deprotonation or nucleophilic attack process.

Later, Xia and coworkers reported a mechanistically similar reaction for the synthesis of perfluoroalkylated aminoquinolines *via* R_f_ radical intermediates (Han et al., 2019) (Figure 3B). The perfluoroalkylation reagents, such as FPIFA, C_6_F_5_I(OCOCF_2_CF_3_)_2_ and C_6_F_5_I(OCOCF_2_CF_2_CF_3_)_2_, were all effective in the reaction. Moreover, similar to reported by Qing et al. (Yang et al., 2018), those HIRs can also be easily re-covered by reaction of the by-product pentafluoroiodobenzene with perfluorocarboxylic acids in the presence of oxone.

Xu and coworkers developed a method of hydrotrifluoromethylation of benzyl-protected homoallylic alcohol and amine derivatives employing Togni's reagent as the CF_3_ radical source under organic photoredox catalysis (Wang et al., 2019) (Figure 3C). Togni's reagent was found to be a more effective trifluoromethylation reagent than CF_3_SO_2_Cl in the reaction. Dye 4CzIPN (2,4,5,6-tetra(9*H*-carbazol-9-yl)isophthalonitrile) has been demonstrated as a competent organic photoredox catalyst for generation of trifluoromethyl radicals from Togni's reagent. It is noteworthy that the reaction proceeds through an oxidative quenching process to deliver a CF_3_· radical followed by a crucial 1,5-hydrogen transfer relay with *in situ* removal of benzyl group.

An efficient photoredox-catalyzed protocol for the introduction of fluorinated groups into the coumarin framework was established by Xiang's group in 2019 (Song et al., 2019) (Figure 3D). The reaction takes place efficiently using *fac*-Ir(ppy)_3_ as the photocatalyst under the irradiation of blue LEDs. When Togni's reagent used as the perfluoroalkylated radical resource in this protocol, *ortho*-hydroxycinnamic esters were converted into 3-trifluoromethylated coumarins *via* a photoredox-catalyzed cascade in moderate to good yields.

### Azidation

Since its first report in 1994 by Zhdankin and co-workers, azidobenziodoxol(on)es (ABXs, Zhdankin reagents) have established themself as valuable alternatives to other azide sources due to easy handling (crystalline solid) and the enhanced stability (being stable up to 130°C) (Fumagalli et al., 2015). These cyclic HIRs have recently been popularly utilized as azide-transfer reagents for azidation of a broad range of substrates (Huang and Groves, 2016). Under visible-light irradiation and in the presence of PC, the weak I–N_3_ bond in azido I(III) reagent frequently undergoes homolytic cleavage to form an azidyl radical and an iodanyl radical, thus triggering the radical chain process to provide the azidated product.

**Figure 4:**
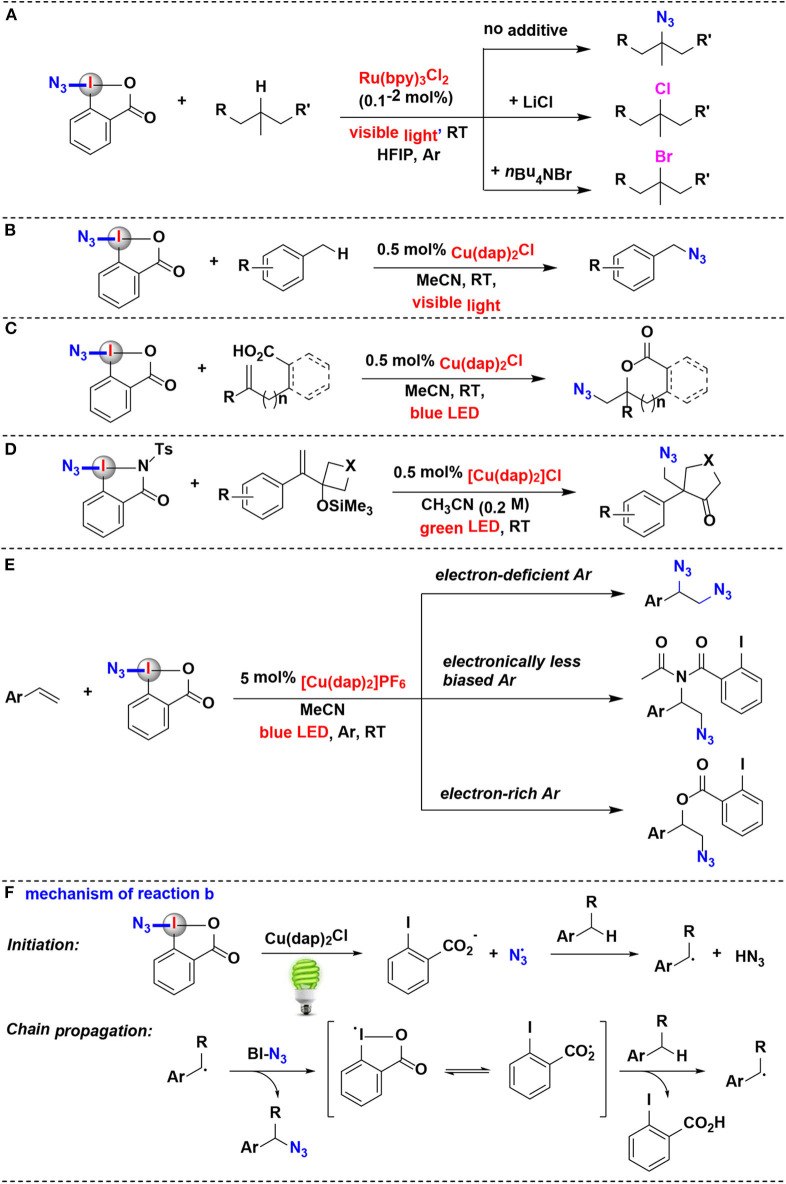
Photoredox-catalyzed azidation using HIRs as azide-transfer reagents. **(A)** Azidation and halogenation of tertiary aliphatic C–H bonds. **(B)** azidation of benzylic C–H bonds. **(C)** Azidation and cyclization of carboxylic acids onto alkenes. **(D)** Azidative ring-expansion of silylated cyclobutanols. **(E)** Azidation/difunctionalization of vinyl arenes. **(F)** Mechanism of reaction **(B)**.

Chen and coworkers disclosed an impressive protocol for the azidation of 3°C(sp^3^)–H bonds of complex substrates using the Zhdankin reagent under Ru photoredox catalysis (Wang et al., 2016) (Figure 4A). The azidation reactions demonstrated excellent 3°C–H selectivity and functional group compatibility. Interestingly, when chlorine or bromide donor was added into the reaction system, this protocol can be further modulated to access aliphatic C–H chlorination and bromination, respectively.

Greaney and coworkers have achieved a direct benzylic C–H azidation using the Zhdankin reagent under photoredox catalysis (Rabet et al., 2016) (Figure 4B). Reaction optimization showed that common photoredox catalysts such as Ru(bpy)_3_Cl_2_ and Ir(ppy)_3_ are totally ineffective, while Sauvage catalyst Cu(dap)_2_Cl is found to be unique for this azidation. Moreover, the C–N bond formation is wide applicable to primary, secondary, or tertiary benzylic position. The authors proposed the reaction mechanism depicted in Figure 4F. It is believed that the photoexcited state ^*^Cu(dap)^2+^ firstly reductive cleaves BI-N_3_ to generate a source of azide radicals, then the azide radical serves as the H abstractor to convert the benzylic C–H substrate to a benzyl radical. Subsequently, the benzyl radical attacks BI-N_3_ to form the azidated product and gives the chain-carrying iodane radical. The iodane radical thus regenerates benzyl radical by abstracting a hydrogen atom from benzylic substrate and then propagates the radical chain reaction.

In 2017, the Waser's group reported a method of synthesis of azidolactones starting from alkene-containing carboxylic acids (Alazet et al., 2017) (Figure 4C). Using Zhdankin reagent as the azide-transfer reagent and only 0.5 mol% Cu(dap)_2_Cl as photoredox catalyst, (1,2)-azidolactones were achieved under visible light irradiation. Zhdankin reagent and azidodimethylbenziodoxole (ADBX), two typical azide-transfer reagents, exhibited divergent reactivity in the azidolactonization: Zhdankin reagent was ideally suited for 1,2-azidation under photoredox conditions, while Lewis acid activation of ADBX led to 1,1-azidolactonization *via* a 1,2-aryl shift. When ADBX was used instead of Zhdankin reagent under the same photoredox conditions, only traces of (1,2)-azidolactones were observed.

Shortly after its discovery, this visible-light-promoted photoredox-catalyzed azidation methodology was elegantly expanded to alkene-substituted cyclobutanol derivatives by the same group (Alazet et al., 2018) (Figure 4D). In 2018, they introduced two new cyclic iodine(III) reagents (CIRs) with higher molecular weight for azidation: *t*Bu-ABX and ABZ (azidobenziodazolone). The two reagents showed a better safety profile than the most commonly used Zhdankin reagent, which was both shock and friction sensitive. Furthermore, either *t*Bu-ABX or ABZ can be used as alternatives to the Zhdankin reagent in a broad range of transformations including photoredox catalysis. They developed an azidative ring-expansion of alkene-substituted cyclobutanol derivatives using ABZ as the safer azido-radical source and Cu(dap)_2_Cl as photoredox catalyst.

In 2019, the group of Yu has investigated the visible-light-driven azidation of vinyl arenes with Zhdankin reagent as azidating agent in acetonitrile by using [Cu(dap)_2_]PF_6_ as photocatalyst (Wu et al., 2019) (Figure 4E). It was found that the electronic nature of the aryl group attached to the olefin moiety plays a profound effect on the reaction consequence: when the aryl group was less electronically biased, amido-azidation products were obtained as major products through a three-component reaction involving the solvent acetonitrile as well as Zhdankin reagent. The mechanistic investigations suggested that these amido-azidation products were probably formed *via* the photoredox catalysis pathway.

### Alkynylation

HIRs, such as alkynyliodonium salts and ethynylbenziodoxol(on)es (EBXs), have been demonstrated as efficient and versatile alkynylating reagents for alkynylation. Very recently, the synergistic merger of photoredox catalysis with HIRs (especially EBXs) paved the way to radical alkynylation of carboxylic acids and alcohols, thus enabling the synthesis of valuable aryl-, alkyl and silyl-substituted acetylenes (Kaschel and Werz, 2015; Waser, 2016; Vaillant and Waser, 2017).

#### Decarboxylative Alkynylation of Carboxylic Acids

**Figure 5:**
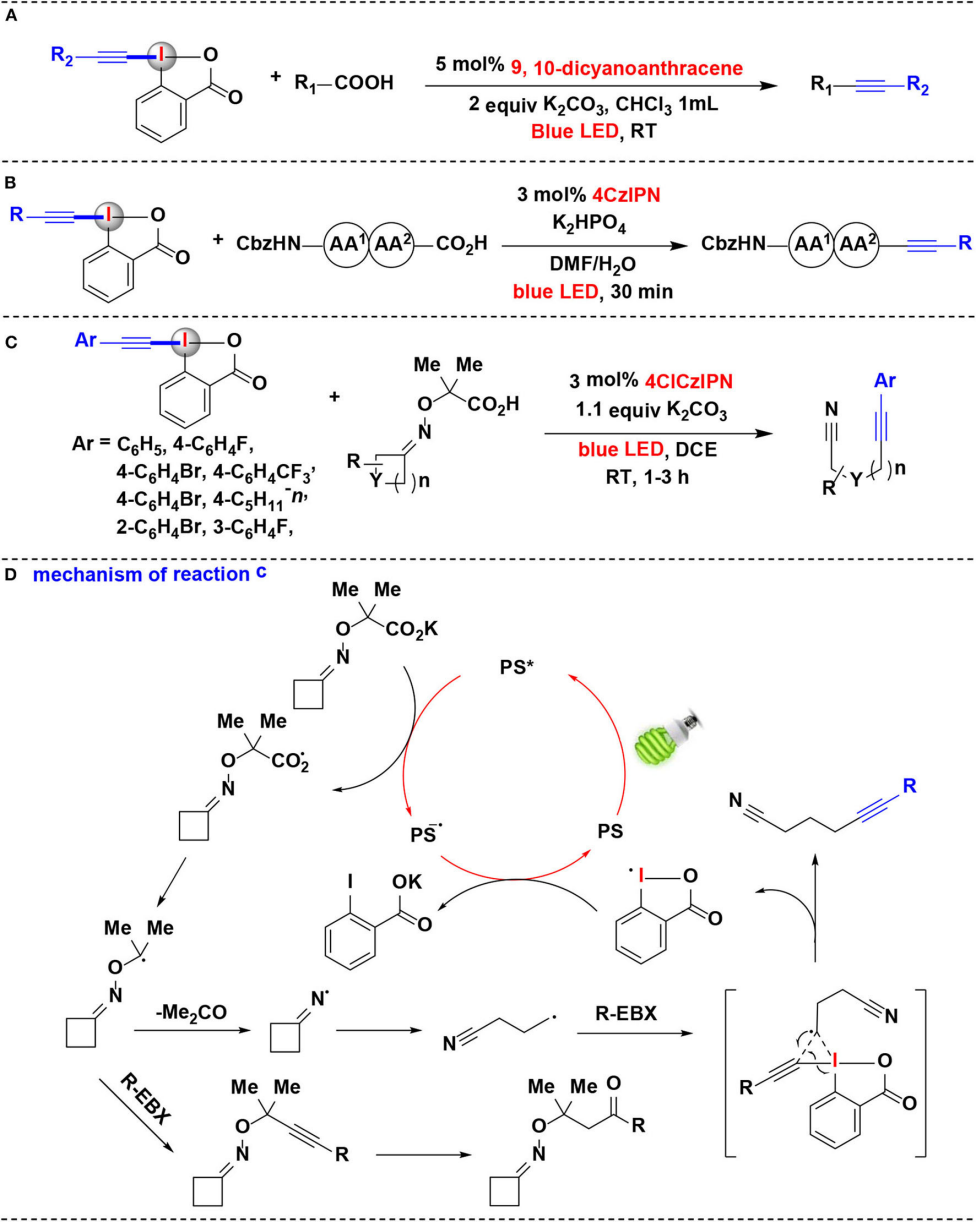
Photoredox-catalyzed decarboxylative alkynylations of carboxylic acids with EBXs. **(A)** Decarboxylative alkynylation of α-amino/α-oxo/α-keto acids. **(B)** Decarboxylative alkynylation of the C-terminus of peptides. **(C)** Fragmentation-alkynylation cascades of cyclic oxime ethers. **(D)** Mechanism of reaction **(C)**.

Based on the previous success on visible-light photoredox catalytic decarboxylative alkynylation of carboxylic acids, Li, Cheng, and co-workers developed a metal-free procedure in which 9,10-dicyanoanthracene (DCA) (Romero and Nicewicz, 2016; Neumeier et al., 2018) serve as the photoredox catalyst for the replacement of the classic iridium catalysts (Yang C. et al., 2016) (Figure 5A). The results showed that carboxylic acids could be efficiently photo-oxidated by only 5 mol% of cheap organic photocatalyst DCA at room temperature. Moreover, natural sunlight can also be used as a light source. A gram-scale reaction further demonstrates the synthetic utility of this methodology.

Due to its mild conditions to generate radicals, the photoredox catalysis provides a rational basis for developing novel strategies in biomolecule functionalization (Hu and Chen, 2015). Especially, photoredox-catalyzed decarboxylation strategies were successfully applied to selectively functionalize the C-terminal position of native peptides. Following their success on photoredox-catalyzed decarboxylative alkynylation of α-amino acids using EBXs, Waser and coworkers recently extended the methodology for decarboxylative alkynylation on C-terminus of peptides (Garreau et al., 2019) (Figure 5B). Using EBXs as alkynylation reagents and 4CzIPN as photoredox catalysts, alkynylated peptides can be efficiently achieved in 30 min at room temperature under blue LEDs irradiation. Moreover, this reaction exhibited superior selectivity for the C-terminus in the presence of carboxylic acid side-chains. The results showed that EBX reagents possess a high potential for biomolecule functionalization under mild photoredox-catalyzed conditions.

In 2018, the same group has shown that EBX reagents allowed the alkynylation of cyclic alkyl ketone oxime ethers through oxidative photoredox cycles, and versatile alkynyl nitriles were synthesized *via* a fragmentation-alkynylation sequence (Franck et al., 2018) (Figure 5C). It is worth noting that modified 4XCzIPN dyes were demonstrated as efficient photoredox organocatalysts in this methodology, and their redox properties were determined by both cyclic voltammetry and computation. Among them, 4ClCzIPN dye exhibited highly efficient in the fragmentation-alkynylation process. Various aryl-substituted EBX reagents worked well under the reaction condition. Preliminary investigations showed that other HIRs, such as silyl EBX reagent (TIPS-EBX), cyanobenziodoxolone (CBX) and phenyl vinyl benziodoxolone (PhVBX), can also react with oxime ethers under the same reaction conditions to achieve the corresponding alkynylation, cyanation, and alkenylation products. However, when Togni's reagent was employed, no desired trifluoromethylation product was obtained.

Based on investigations conducted in this study, it is believed that the mechanistic pathway in this process (Figure 5D) begins with reductive quenching of the photoexcited state PS^*^ of 4ClCzIPN dye by potassium carboxylate to give carboxyl radical and the reduced state photocatalyst. The resulting carboxyl radical undergoes decarboxylation to furnish the α-oxy radical, which subsequently eliminates the acetone to generate iminyl radical. ^1^H NMR evidence showed that the carboxyl radical can also be trapped by EBX reagent and then hydrated to give a by-product of the ketone. Ring-opening of the iminyl radical then gives an alkyl nitrile radical. The alkyl nitrile radical reacts with EBX and proceeds through a transition state to give the final product and cyclic hypervalent iodine radical. The reduction of the hypervalent iodine radical provides carboxylate and regenerates the ground state PS to accomplish the organocatalysis cycle.

#### Alkynylation of Alcohols

Similar to carboxylic acids, alcohols can also be efficiently alkynylated employing EBXs as alkynylating reagents under photoredox-catalyzed conditions. It should be noted that an HIR catalysis circle, in which HIR catalyzes the generation of alkoxyl radicals, is often combined with the photoredox catalysis circle in those methodologies.

**Figure 6:**
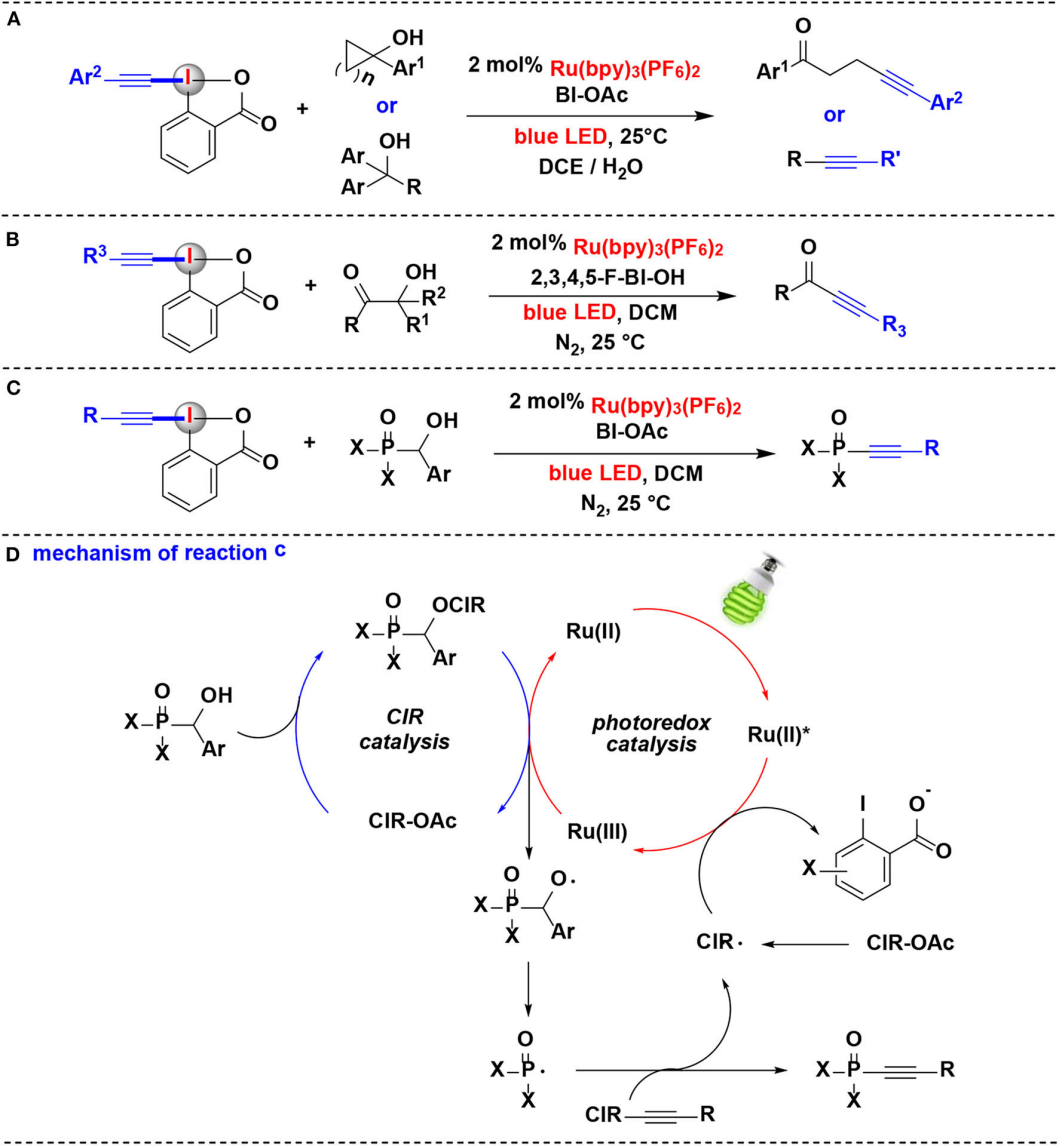
Photoredox-catalyzed alkynylation of alcohols with EBXs. **(A)** Alkynylation of cycloalkanols and linear alcohols. **(B)** Alkynylation of β-amide/β-ester/β-ketone alcohols. **(C)** Alkynylation of a-phosphorus alcohols. **(D)** Mechanism of reaction **(C)**.

Chen and co-workers have conducted a series of studies aiming at photoredox-catalyzed alkynylation of different types of alcohols. In 2016, this group exploited the combination of photoredox catalysis and CIR catalysis for alkynylation of alcohols using alkyl-EBX reagents (Jia et al., 2016) (Figure 6A). Under the dual CIR/photoredox catalytic system, both strained cycloalkanols and linear alcohols can react with alkyl-EBXs delivering the corresponding alkynylation adducts. Moreover, structurally complex steroidal cycloalkanols can also convert into χ-alkynyl ketones smoothly. Various aryl substituents appended to EBXs are suitable for this process. The key to success in this transformation was the visible-light-induced alcohol oxidation for generation alkoxyl radicals and the subsequent β-fragmentation of alkoxyl radicals into alkyl radicals. Compared with those that employ transition metal activation under strong oxidative conditions, visible-light-induced alkoxyl radical generation by CIR catalysis proceeds smoothly at room temperature.

In 2017, this group also developed another C-C bond cleavage/alkynylation reactions of β-amide, β-ester, and β-ketone alcohols with EBXs *via* similar dual CIR/photoredox catalysis, and ynamides, ynoates, and ynones were respectively constructed with excellent regio- and chemoselectivity (Jia et al., 2017) (Figure 6B).

Following the above successes, they further extended the dual CIR/photoredox catalytic methodology to α-phosphorus alcohols in 2018 (Jia et al., 2018) (Figure 6C). Various arylphosphinoyl-, alkylphosphinoyl-, phosphonate-, and phosphonic amide alcohols undergo P-C(sp^3^) bond cleavage/radical alkynylation with EBXs to construct phosphonoalkynes for the first time. Different cyclic iodine(III) reagents, such as BIOAc, 3,4-F-BIOAc, 2,3,4,5-F-BIOH, and 3,4-OMe-BIOAc, were all effective to promote the reaction. A range of EBXs (BI-alkynes) including o*rtho*-, *meta*-, or *para*-aryl substituents were well tolerated in the reaction.

A plausible mechanism for this process is depicted in Figure 6D, the α-phosphorus alcohol first reacted with CIR to generate the benziodoxole/α-phosphorus alcohol complex *in situ*, which releases the alkoxyl radical and revives of CIR for the new catalytic cycle upon oxidation by Ru(bpy)${}_{3}^{3+}$. The Ru(bpy)${}_{3}^{3+}$ was originated from the oxidative quenching of the photoexcited ^*^Ru(bpy)${}_{3}^{2+}$ by CIR. The resulting alkoxyl radical subsequent carries on P-C(sp^3^) bond cleavage to generate the phosphorus radical, and further performs radical α-addition with the BI-alkyne to yield the desired phosphonoalkyne product.

### Other Reactions

#### Cyanation

**Figure 7:**
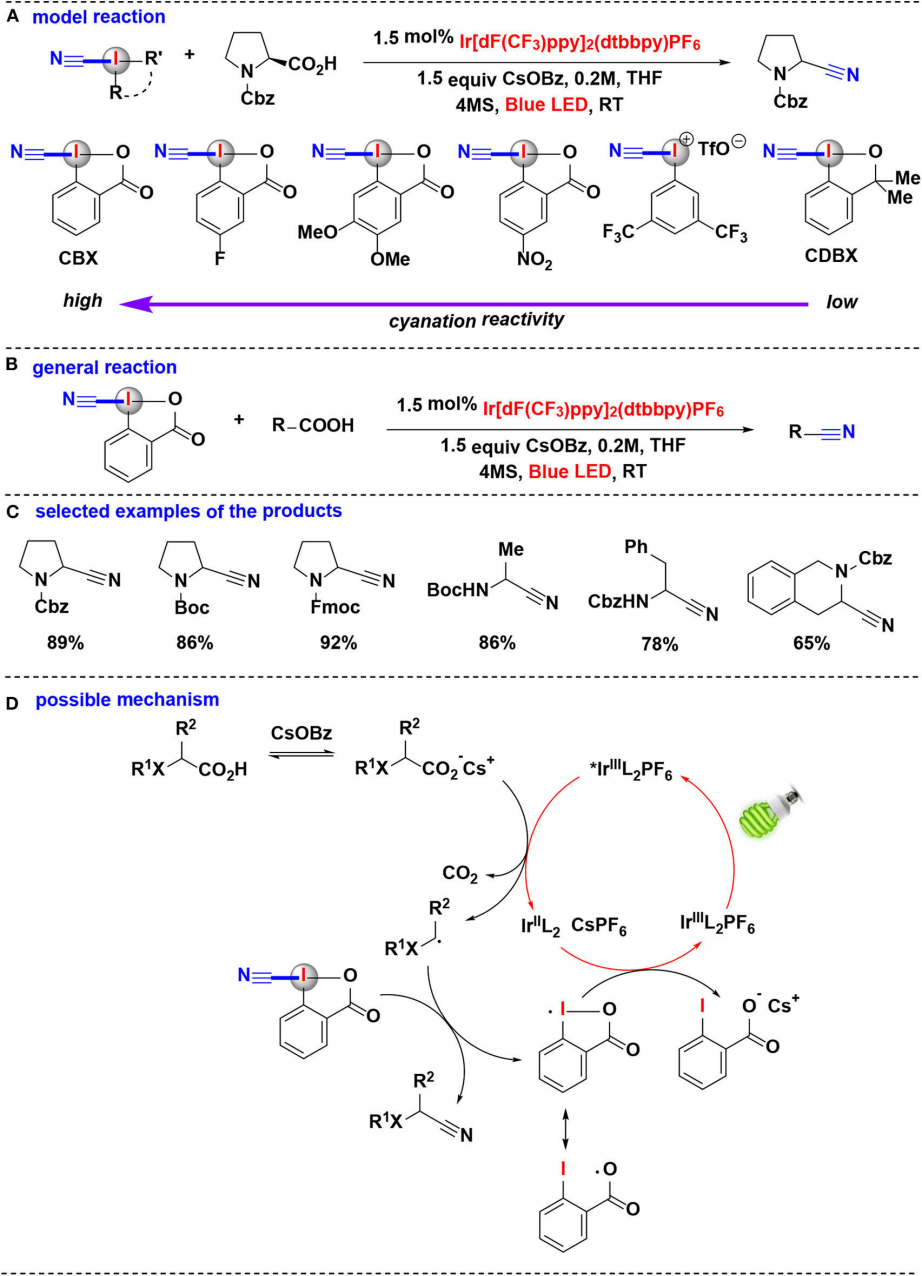
Photoredox mediated decarboxylative cyanation of carboxylic acid with CBX. **(A)** Model reaction of decarboxylative cyanation. **(B)** General reaction of decarboxylative cyanation. **(C)** Selected examples of the cyanation products. **(D)** Possible mechanism.

In 2017, Waser's group extensively investigated the photoredox mediated decarboxylative cyanation of aliphatic acids using HIRs as cyano-transfer reagents (Le Vaillant et al., 2017) (Figure 7). In their model reaction, the cyanation reactivities of six hypervalent iodine-based cyanation reagents were evaluated (Figure 7A). Under photoredox catalysis, CDBX and acyclic iodine reagent were almost inefficient while cyanobenziodoxolone (CBX) gave the product in excellent yield, these results showed the superiority of CBX as a cyanide source. The subsequent substrate scope investigation indicated that this methodology allowed efficient cyanation of α-amino and α-oxy acids into the corresponding nitriles (Figures 7B,C). Furthermore, the direct cyanation of dipeptides and drug precursors was also achieved.

Computational and experimental evidences suggested that the favored decarboxylative cyanation mechanism may probably different from the usually assumed decarboxylative alkynylation (Le Vaillant et al., 2015; Zhou et al., 2015). The proposed reaction mechanism (Figure 7D) consists of the irradiation of IrL${}_{2}^{+}$ with blue LED gives the excited-state ^*^IrL${}_{2}^{+}$, which subsequently carries on SET process with the *in situ* generated cesium carboxylate to regenerate the IrL_2_ complex and together give the key nucleophilic radical intermediate. The reaction of the radical intermediate with CBX provides the desired nitrile and an iodine centered radical. Finally, this iodine centered radical undergoes another SET process with the IrL_2_ complex to close the catalytic cycle.

#### Acetoxylation

**Figure 8:**
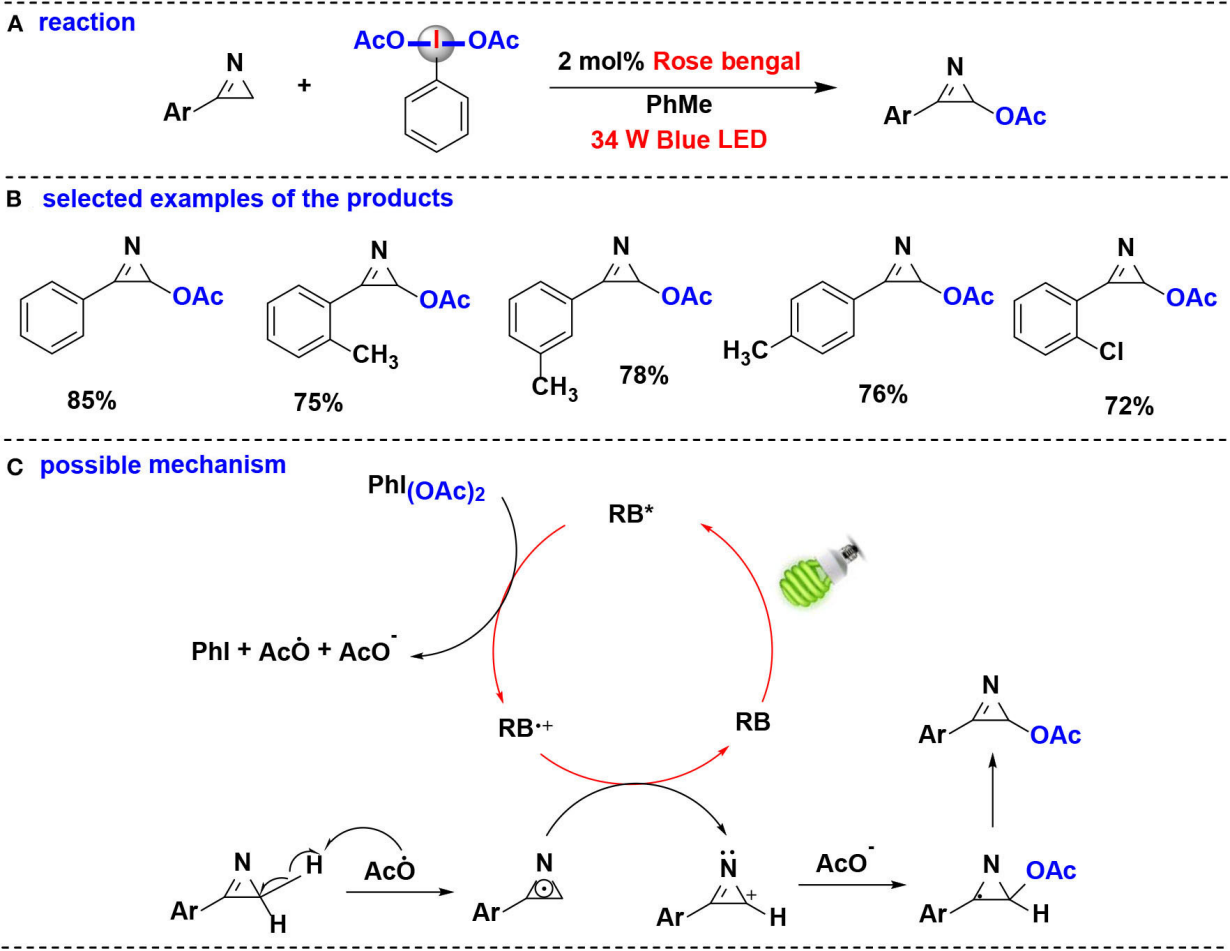
Photoredox-catalyzed C(sp^3^)-H acetoxylation of aryl-2*H*-azirines with PIDA. **(A)** General acetoxylation reaction of aryl-2H-azirines. **(B)** Selected examples of the products. **(C)** Possible mechanism.

In 2019, Santra, Hajra, Majee and coworkers developed a method for regioselective coupling of C(sp^3^)-H of aryl-2*H*-azirine and (diacetoxy)-iodobenzene (PIDA) using visible light irradiation (De et al., 2019) (Figure 8). Aryl-2*H*-azirines with different functional groups were converted into the corresponding acetoxylated azirines under aerobic condition. Organophotocatalyst, rose Bengal (RB), was found to be more efficient in this reaction than transition-metal photoredox catalysts, such as Ru(bpy)_3_Cl_2_·6H_2_O and Ir(ppy)_3_. Notably, this protocol can be carried out in gram-scale.

The proposed mechanism of the acetoxylation reaction is shown in Figure 8C. Firstly, when irradiation with blue LED, rose bengal (RB) was excited into the excited state RB^*^, which performs an SET reduction with PIDA to generate the acetoxy radical (CH_3_COO·), accompanied by formation of the cation radical (RB^+·^), PhI, and CH_3_COO^−^. Abstraction of the hydrogen atom of aryl-2H-azirine by acetoxy radical provides the 2*H*-azirine radical. The 2*H*-azirine radical then undergoes a second SET oxidation with RB^+·^, leading to the formation of intermediate carbocation while completing the photocatalytic cycle. Finally, the intermediate carbocation couples with the acetate anion giving the corresponding acetoxylated azirine.

#### Diazomethylation

**Figure 9:**
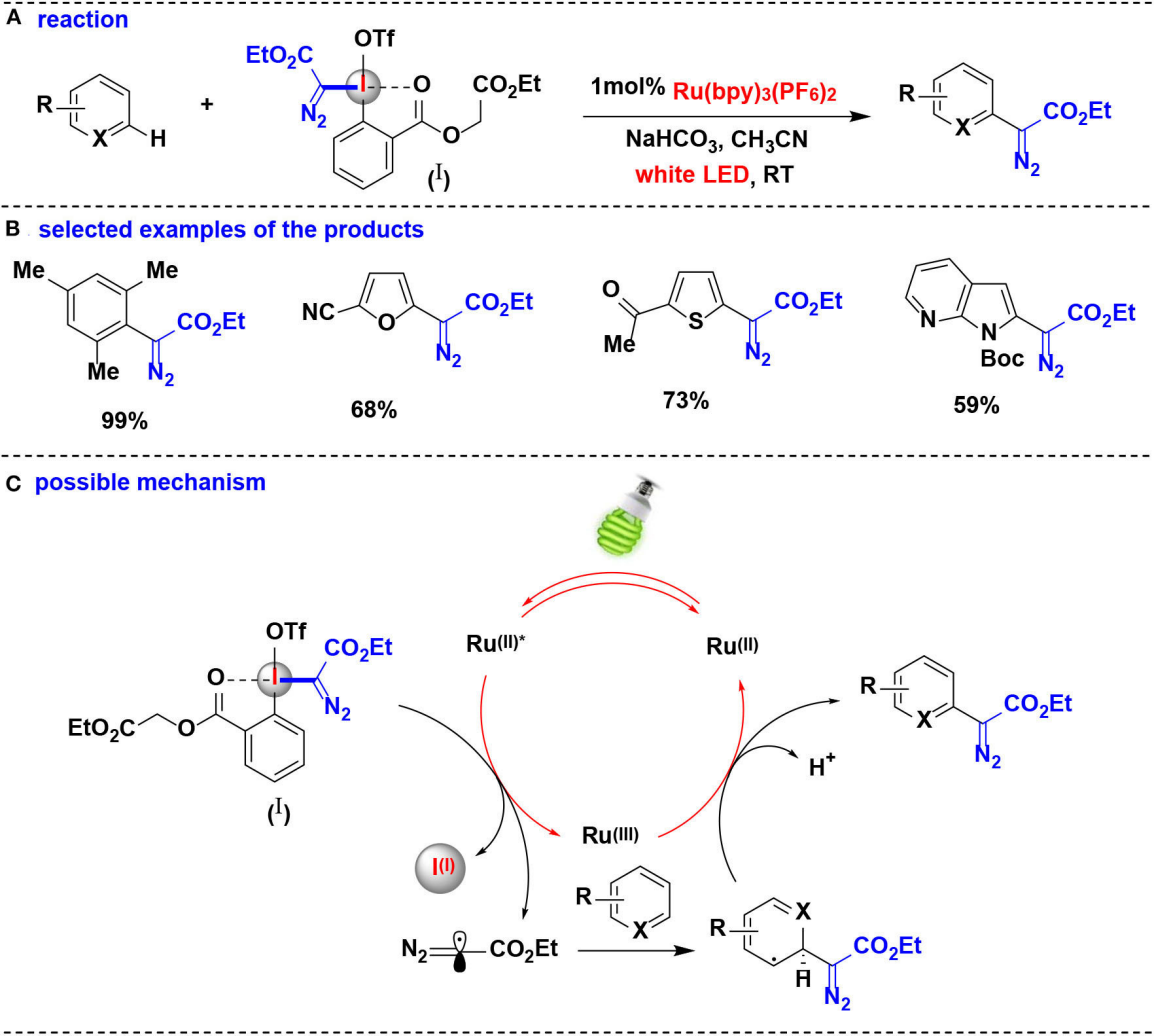
Photoredox catalysis enabled C–H bond diazomethylation of arenes with pseudocyclic HIR. **(A)** General diazomethylation reaction of arenes. **(B)** Selected examples of the products. **(C)** Possible mechanism.

In 2018, Suero and co-workers developed an aromatic C-H bond diazomethylation reactions using the pseudocyclic hypervalent iodine (I) by ruthenium photoredox catalysis (Wang Z. et al., 2018) (Figure 9). The pseudocyclic hypervalent iodine (I) carrying a diazoacetate moiety served as a diazomethyl radical precursor through a SET process in photoredox-catalyzed protocol, and a wide range of aromatic hydrocarbons substituted with alkyl groups, halogens, amides and carbonyls undergo C-H diazomethylation to generate valuable diazo compounds.

The authors proposed the reaction mechanism depicted in Figure 9C. The photocatalytic system is initiated by the photoexcitation of [Ru(bpy)_3_]^2+^ to generate ^*^[Ru(bpy)_3_]^2+^. The photoexcited ^*^[Ru(bpy)_3_]^2+^ undergoes single-electron transfer with the pseudocyclic hypervalent iodine (I) to yield the diazomethyl radical as direct equivalent of carbyne specie, which is further intercepted an aromatic ring to facilitate the cyclohexadienyl radical formation. Finally, the resulting radical intermediate is oxidized by [Ru(bpy)_3_]^3+^ and eliminates the proton to obtain the expected diazo compound.

## HIRs Act as Oxidants for Substrate Activation

Due to the excellent coordinating property of iodine atom, HIRs can easily experience ligand exchange reaction with organic acids to form the hypervalent iodine-coordinated carboxylates. When combination with the photoredox catalysis, those hypervalent iodine-coordinated carboxylates frequently undergo homolytic cleavage to access highly reactive hypervalent iodine radicals as well as the oxygen radicals, thus triggering the decarboxylative functionalization reactions or other transformations (Huang et al., 2016; Jia et al., 2018). Based on the above concept, Chen and co-workers have conducted a series of studies on novel dual CIR/photoredox catalytic system (Huang et al., 2015; Jia et al., 2016, 2017), and the research results proved that CIRs played a crucial role in activating the substrates of organic acids and alcohols toward photoredox catalysis.

### HIR-Mediated Activation of Organic Acids

**Figure 10:**
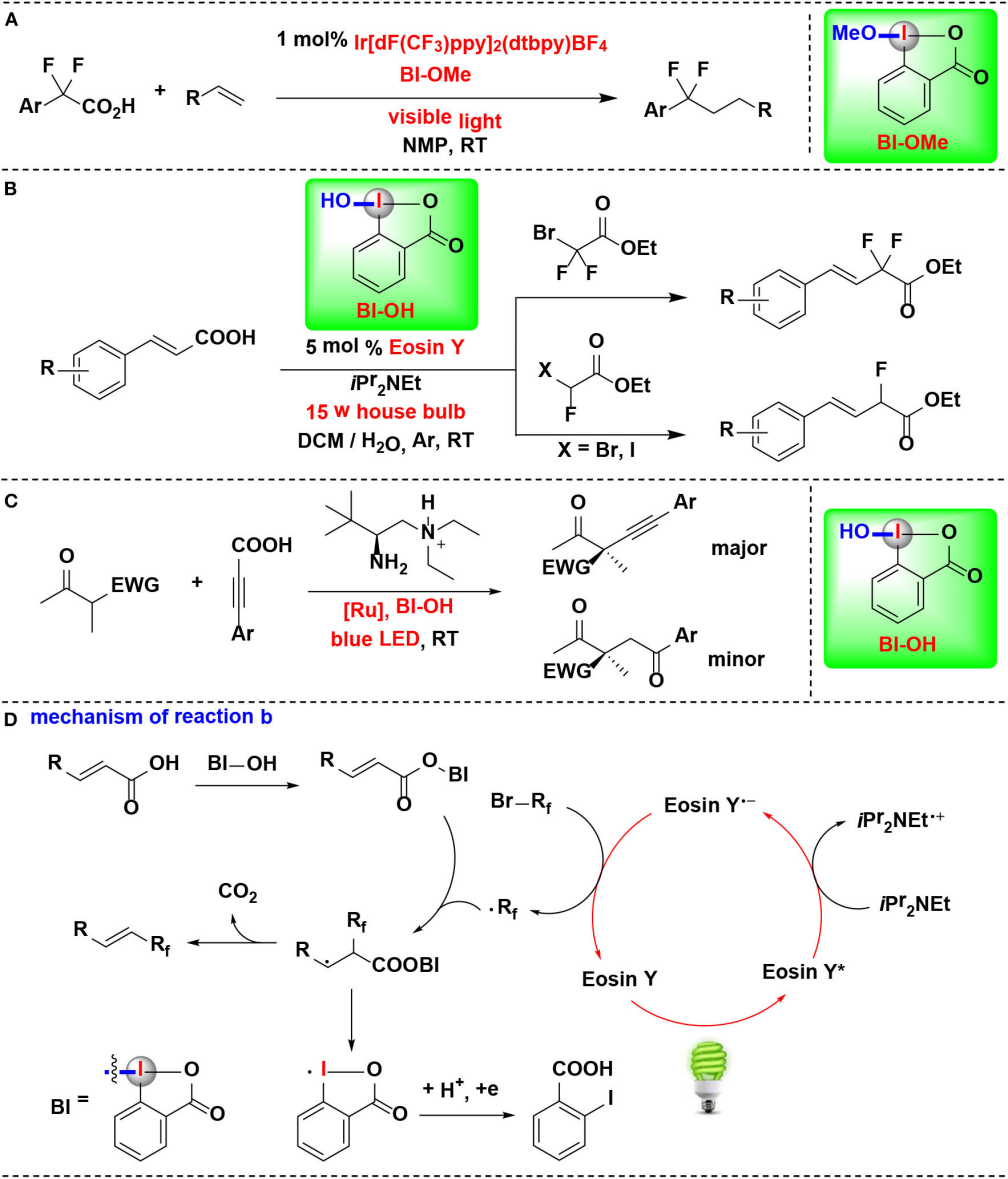
HIR-mediated activation of α,α-difluoroarylacetic acids, α,β-unsaturated carboxylic acids and propiolic acid under photoredox catalysis. **(A)** Activation of α,α-difluoroarylacetic acids for hydroaryldifluoromethylation of alkenes. **(B)** Activation of α, β-unsaturated carboxylic acids for decarboxylative fluoromethylation. **(C)** Activation of propiolic acids for decarboxylative α-alkynylation. **(D)** Mechanism of reaction **(B)**.

An example of CIR-enabled decarboxylative functionalization of α, α-difluoroarylacetic acids, mediated by dual CIR/photoredox catalysis, were developed by Qing and coworkers (Yang B. et al., 2016) (Figure 10A). A series of novel difluoroalkylated arenes were smoothly achieved through an HIR-promoted decarboxylation and radical hydroaryldifluoromethylation sequence. All of the tested HIRs including PhI(OAc)_2_, PhI(OCOCF_3_)_2_, BIOAc and BIOMe give the desired transformation. Among them, BIOMe was the best choice. Further investigation revealed that BIOMe acts not only as an activating reagent but also as an oxidant in the process.

Feng, Xu, and coworkers disclosed a visible-light-enabled reaction in which α,β-unsaturated carboxylic acids are activated by BI-OH, thus leading to the decarboxylative mono- and difluoromethylation transformations (Figure 10B) (Tang et al., 2017). Four candidate HIRs, IBDA, IB, BI-OH, BI-OAc, were screened in the reaction. Among them, BI-OH turned out to be optimal. As explained in mechanistic pathway (Figure 10D), BI-OH can *in sute* generate a benziodoxole vinyl carboxylic acid complex (BI-OOCCH=CHR), thus activating of the vinyl carboxylic acid group.

Zhang, Luo, and coworker achieved enantioselective decarboxylative coupling of propiolic acid and β-ketocarbonyls by combination of chiral primary amine catalysis and visible-light photoredox catalysis (Figure 10C) (Wang et al., 2017). Various of alkynylation adducts were synthesized with excellent enantioselectivities under mild conditions. For HIRs tested in this process, PIFA, PIDA, BI-OAc, and BI-OMe performed almost no catalysis effect, and BI-OH were identified to give the optimal results in terms of both yield and enantioselectivity. Mechanistic studies revealed that BI-OH could *in situ* react with propiolic acid to generate the propiolate under the reaction conditions. This propiolate acted as a key intermediate both in photoredox catalytic circle and the aminocatalytic circle.

**Figure 11:**
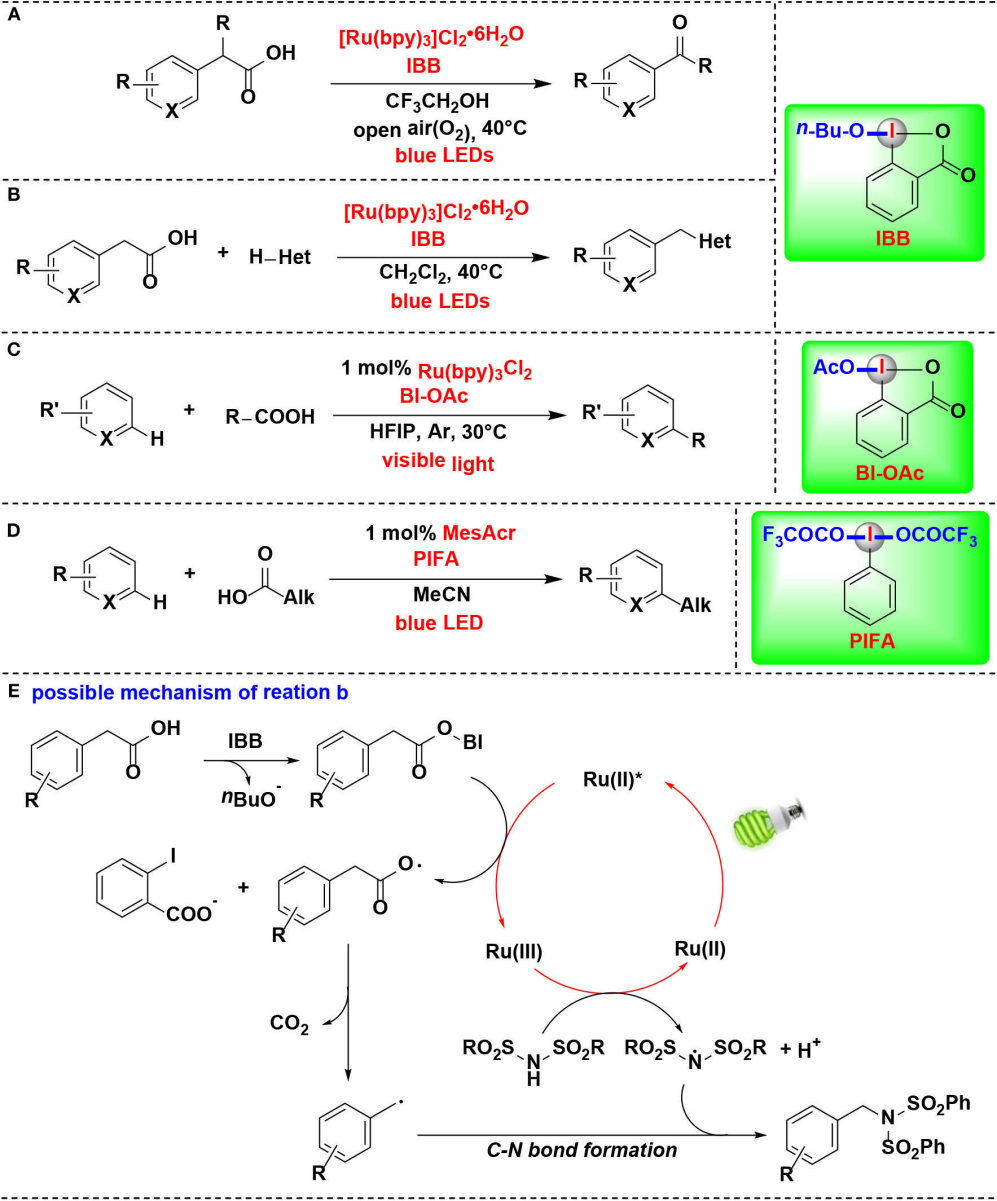
HIR-mediated activation of carboxylic acids under photoredox catalysis. **(A)** Activation of arylacetic acids for decarboxylative oxidation. **(B)** Activation of arylacetic acids for decarboxylative C-X bond formation. **(C)** Activation of aliphatic carboxylic acids for Minisci alkylation. **(D)** Activation of carboxylic acids for C–H alkylation. **(E)** Possible mechanism of reaction **(B)**.

Itami and co-workers developed a mild method for the photoredox-catalyzed decarboxylation of arylacetic acids by HIR in air, thus leading to various aryl-aldehydes and ketones (Sakakibara et al., 2018a) (Figure 11A). Photoredox catalyst, HIR, blue light irradiation, and O_2_ are all critically important for this transformation. CIR 1-butoxy 1-λ^3^-benzo[*d*][1,2]iodaoxol-3(1*H*)-one (IBB) was proved more efficient in the procedure than non-cyclic iodine reagent PIDA. In contrast, Ph_2_ICl was completely inefficient. In this process, IBB reacts with arylacetic acid to form intermediate *in situ*, thus activating of the arylacetic acid for decarboxylation.

The same group's subsequent study revealed that the same methodology can also be extended for construction of carbon–nitrogen and carbon–oxygen bonds (Figure 11B) (Sakakibara et al., 2018b). Under the activation of IBB, arylacetic acids were directly converted into nitrogen, oxygen, or chlorine functionalities. The reaction of IBB with arylacetic acid was confirmed by ^1^H NMR, and the resulting complex was a key activated intermediate in the photoredox catalytic cycle of the mechanism pathway.

The authors raised a possible mechanism for the decarboxylative imidation (Figure 11E). Initially, arylacetic acid reacts *in situ* with IBB to form benziodoxole/arylacetic acid complex. Meanwhile, the photocatalyst ([Ru(bpy)_3_]^2+^) is excited under irradiation of blue light to generate its photoexcited state (^*^[Ru(bpy)_3_]^2+^). Then the excited ruthenium photocatalyst reduces the benziodoxole/arylacetic acid complex to give [Ru(bpy)_3_]^3+^, arylacetic radical, and *o*-iodobenzoate. The arylacetic radical in turn suffers decarboxylation to produce benzyl radical. Parallel to this process, another substrate, imide, is oxidized by [Ru(bpy)_3_]^3+^ to provide imidyl radical. Finally, radical-radical coupling of the arylacetic radical and imidyl radical affords the imidation product.

In 2018, the Chen's group further expanded their protocol of photoredox-mediated Minisci alkylation of *N*-heteroarenes reported in 2016 (Li G. X. et al., 2016). In the improved protocol (Figure 11C) (Wang J. et al., 2018), the alkylating agents were replaced by aliphatic carboxylic acids, which are more abundant, inexpensive, stable and structurally diverse than alkyl boronic acids. Although the same HIR was employed in both protocols, it actually demonstrated different roles under the photoredox catalysis conditions, and these two reactions proceed through different mechanisms. BI-OAc serves as a radical precursor in former, while in the improved protocol, it is used for substrate activation to facilitate decarboxylative functionalization of carboxylic acids.

Genovino, Frenette, and coworkers developed a C–H alkylation of heteroaromatics using an acridinium photocatalyst and HIRs (Figure 11D) (Genovino et al., 2018). Bis(trifluoroacetoxy)iodo benzene (PIFA), a more soluble and under-utilized HIR, was proved as attractive option. It is noteworthy that the more challenging linear carboxylic acids that form primary radicals are also suitable substrates. A mechanism pathway, which different from other photoredox Minisci reactions catalyzed by transation-metals, was proposed by the authors.

**Figure 12:**
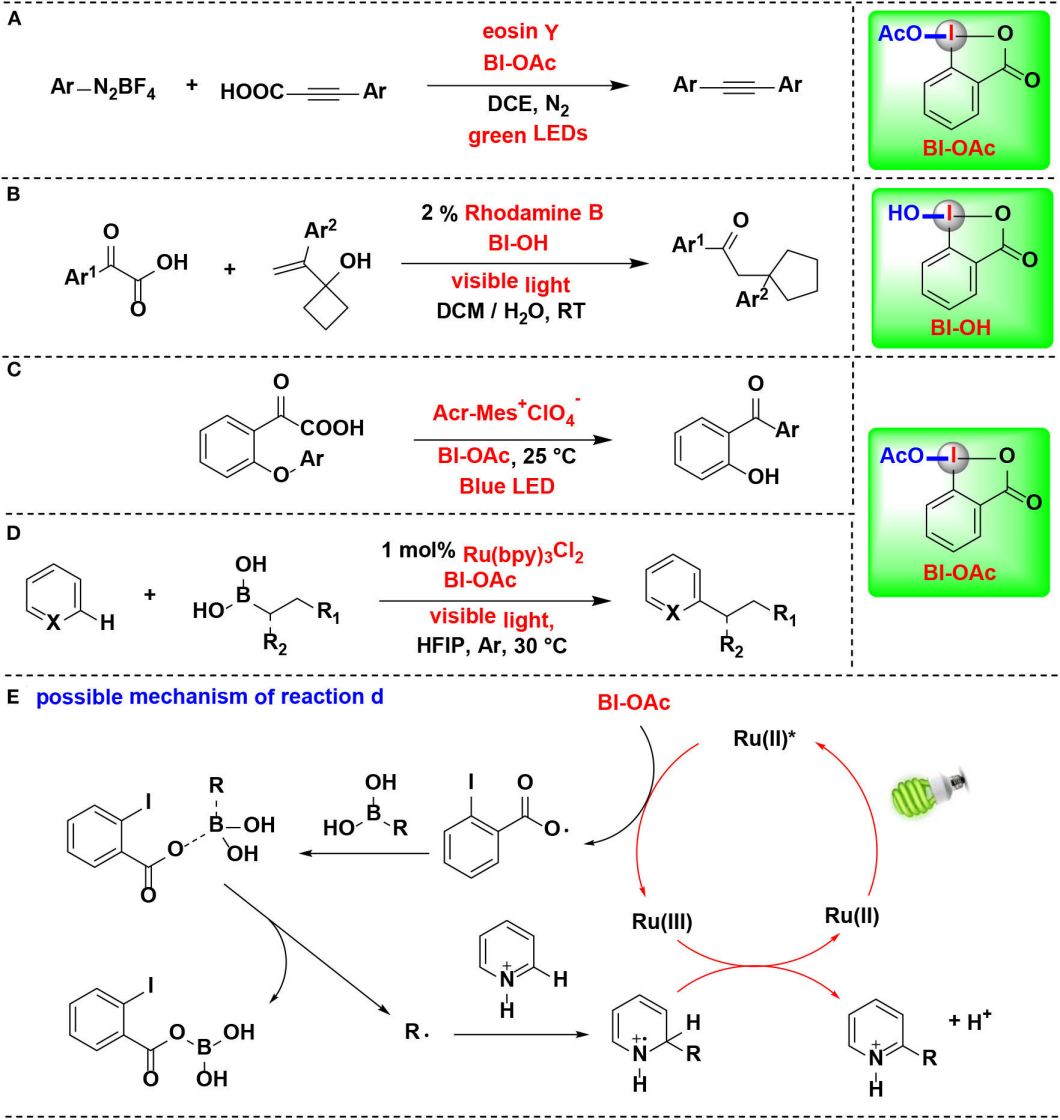
HIR-mediated activation of arylpropiolic acids, α-keto acid and boronic acids under photoredox catalysis. **(A)** Activation of arylpropiolic acids for decarboxylative alkynylation. **(B)** Activation of α-keto acids for decarboxylative acylation/ring expansion. **(C)** Activation of α-keto acids for acyl Smiles rearrangement. **(D)** Activation of alkyl boronic acids for Minisci C–H alkylation. **(E)** Possible mechanism of reaction **(D)**.

In 2019, Cheng reported a decarboxylative coupling of alkynyl carboxylic acids and aromatic diazonium salts using HIR under eosin Y photoredox catalysis (Figure 12A) (Yang et al., 2019). The results showed that BI-OAc superior to BI-OH and BIOMe as decarboxylation facilitated reagent for the reaction. BI-OAc and arylpropiolic acid generated a benziodoxole 3-phenylpropiolate complex *in situ*, which facilitated C–C triple bond conversion in the mechanical pathway proposed by the authors.

Duan and coworkers reported the decarboxylative acylation/ring expansion reactions between vinylcyclobutanols with α-keto acids to construct 1,4-dicarbonyl compounds (Figure 12B) (Zhang et al., 2017). This methodology takes advantage of organic photoredox catalysis and merges it with HIR. Both transition-metal and organic photoredox catalysts were examined in the reaction, among them, rhodamine B, an organic dye known for its low cost, less toxic and easy to handle, give the best results. BI-OH was proved to play an important role in facilitating decarboxylation of α-keto acids. Radical-trapping experiments confirmed that nucleophilic acyl radical, which originated from α-keto acid, was involved in this tandem radical process.

Chen and coworkers developed the first acyl radical Smiles rearrangement for transformation of biarylethers into hydroxybenzophenones (Li J. et al., 2019) (Figure 12C). Under dual hypervalent iodine(III)/photoredox catalysis, α-keto acids undergo ester exchange with BI-OAc to form BI-keto acid complexes *in situ*, which can readily afford acyl radicals and then suffer 1,5-ipso addition and eventually give hydroxybenzophenones. Two typical non-cyclic iodine(III) reagents, PIDA and PIFA, were proved both less effective than BI-OAc. Organic photocatalyst 9-mesityl-10-methylacridinium perchlorate (Acr-Mes^+^ClO${}_{4}^{-}$) superiors to [Ru(bpy)_3_](PF_6_)_2_ and [Ir(ppy)_2_(dtbbby)]PF_6_ and gives optimal yields. Particularly, the reaction can be applied in gram-scale synthesis and performed in neutral aqueous conditions, implying its potential biomolecule applications in further.

In 2016, Chen and co-workers developed a new photoredox-mediated protocol for Minisci C–H alkylation of *N*-heteroarenes using alkyl boronic acids as alkylation regents, BI-OAc as oxidants, and Ru(bpy)_3_Cl_2_ as photocatalyst (Li G. X. et al., 2016) (Figure 12D). This protocol can be applicable to a range of easily accessible primary and secondary alkyl boronic acids for the preparation of various *N*-heteroarenes, and various functional groups, including alkyl bromide, aryl iodide, ester, amide, carbamate, terminal alkyne, and benzyl chloride, are well-tolerated. Mechanistic experiments suggested that BI-OAc serves as a facile precursor for an ortho-iodobenzoyloxy radical intermediate, which play a key role in the efficient transformation of usually less reactive alkyl boronic acids to form alkyl radicals (Figure 12E).

### HIR-Mediated Activation of Alcohols

**Figure 13:**
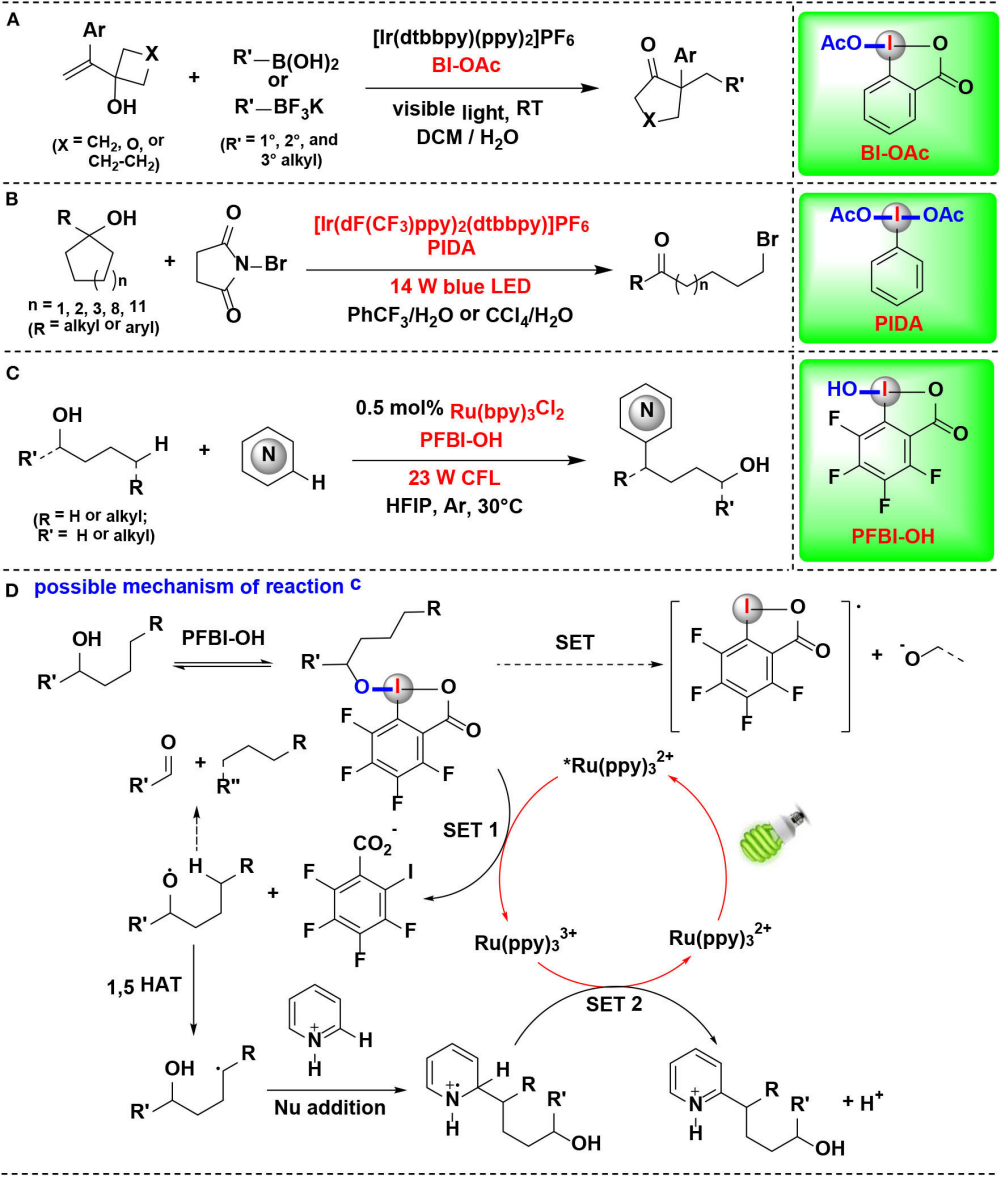
HIR-mediated activation of alcohols under photoredox catalysis. **(A)** Activation of allylic alcohols for alkyl boronate addition/rearrangement. **(B)** Activation of unstrained cycloalkanols for ring-opening bromination. **(C)** Activation of aliphatic alcohols for remote C–H heteroarylation. **(D)** Possible mechanism of reaction **(C)**.

Chen and coworkers reported in 2018 that allylic alcohols can be activated by CIRs under photoredox catalysis conditions, and a series of cyclopentanones, cyclohexanones, and dihydrofuranones bearing α-quaternary centers were synthesized *via* alkyl boronate addition/semi-pinacol rearrangement (Figure 13A) (Liu et al., 2018). The interaction between tertiary allylic alcohol and BI-OAc was extensively investigated by crystallography, NMR spectroscopy and cyclic voltammetry experiments, and the results revealed that both the hydroxyl and olefin groups in allylic alcohols were greatly activated *via* coordination to the BI-OAc. The mechanistic investigations suggest that the CIRs employed in this reaction played at least triple roles in the whole pathway: (1) facilitating the formation of the alkyl radical and the cation intermediate, (2) activating the allylic alcohol, and (3) the *in situ* protecting of alcohols for avoiding the formation of the epoxide.

Mao, Zhu, and coworkers reported the synthesis of distal bromo-substituted alkyl ketones by visible light-promoted ring-opening functionalization of unstrained cycloalkanols (Wang D. et al., 2018) (Figure 13B). A set of medium- and large-sized rings, such as cyclopentanols, cyclohexanols, cycloheptanols, cyclododecanols, and cyclopentadecanols, are readily brominated through inert C–C bond scission with the assistance of HIR under visible-light irradiation. HIRs such as PIDA, BI-OH, IBX, and DMP were all effective for the reaction, and PIDA gave the best results. Two pathways were proposed for the formation of the key alkyloxy radical by authors. In one of them, PIDA was transesterificated with cycloalkanol *in situ*, thus facilitating generation of the challenging alkoxyl radical.

In 2019, Chen and coworkers discovered a method for δ C(*sp*^3^)–H heteroarylation of free aliphatic alcohols with various *N*-heteroarenes using HIRs as oxidant under Ru photoredox catalysis (Li G. X. et al., 2019) (Figure 13C). Both cyclic I(III) reagents (BI-OAc, BI-OH, PFBI-OH and PFBI-OAc) and acyclic I(III) reagents (PIDA and PIFA) were examined and PFBI-OH achieved the highest efficiency. The high electrophilicity of the iodo center of PFBI-OH makes itself more electrophilic for alcoholysis and easily reducible in SET process. Notably, this method also possesses the advantage of avoiding the use of a large excess of alcohols.

The heteroarylation process (Figure 13D) starts with *in situ* alcoholysis of PFBI-OH with alcohol, and then an alkoxy radical intermediate is generated through the SET reduction. Subsequently, the alkoxyl radical intermediate undergoes 1,5-Hydrogen atom transfer (1,5-HAT) to generate C-radical, which is then engaged in Minisci-type C–C bond formation to give heteroaryl cation intermediate. Finally, the intermediate is converted into target heteroarene through SET oxidation process.

### HIR-Mediated Activation of Alkyl C-H Bonds

Chen Gong and coworkers have conducted a series of studies using HIRs as oxidants to selective functionalization of alkyl C(*sp*^3^)-H bonds under photoredox-catalysis. In these HIR-mediated methods, unactivated alkyl C(*sp*^3^)-H bonds, such as tertiary, benzylic methylene, methylene, and methyl C-H bonds, can be selectively cleaved by benziodoxole radicals (BI·), thus offering straightforward methodologies to synthesis of complex alkyl-substituted compounds from a wide range of acyclic alkanes.

**Figure 14:**
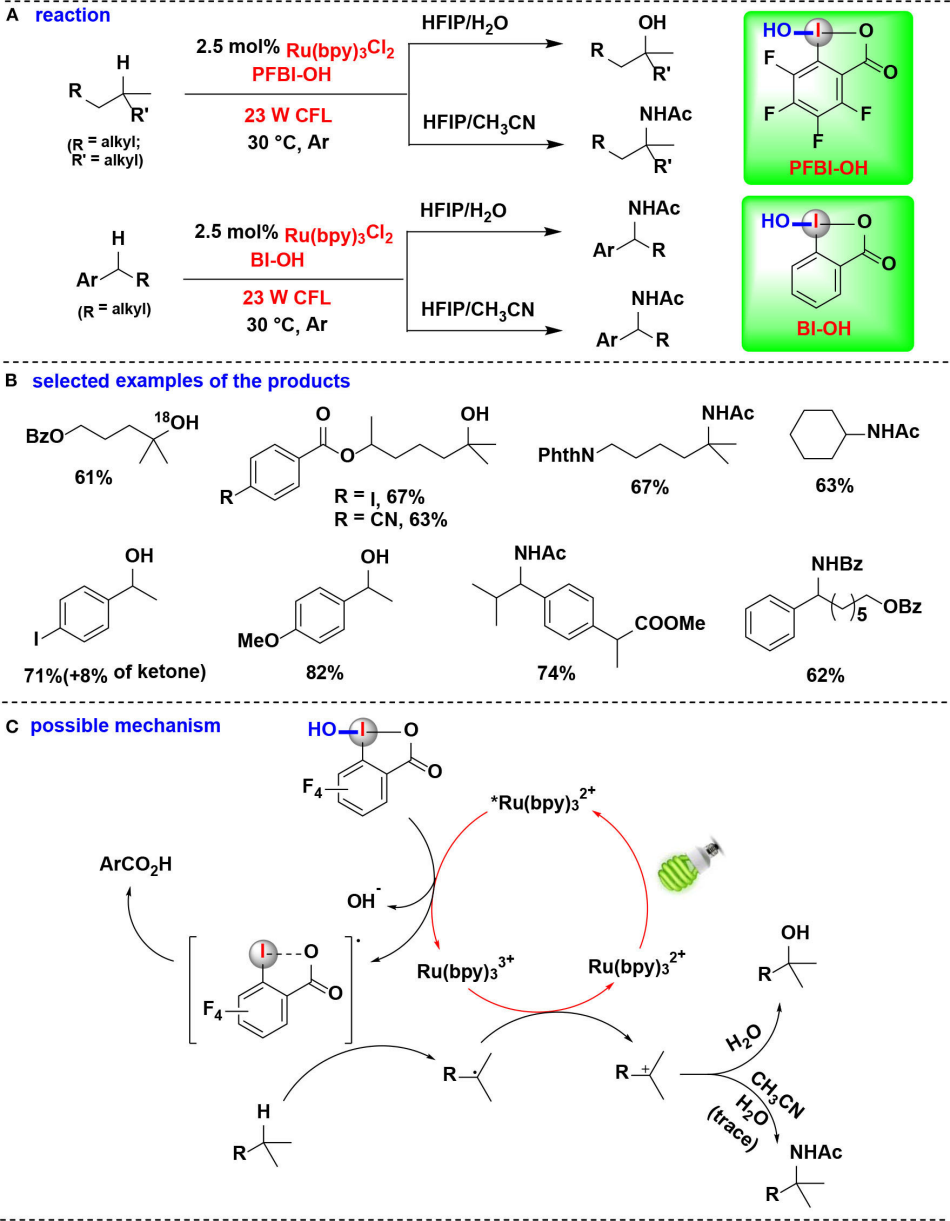
Photoredox-catalyzed C(sp^3^)–H hydroxylation and amidation. **(A)** Activation of tertiary and benzylic C–H bonds for hydroxylation and amidation. **(B)** Selected examples of the products. **(C)** Possible mechanism.

In 2017, this group demonstrated the use of HIRs in both hydroxylation and amidation of tertiary and benzylic C–H bonds, enabled by their corresponding benziodoxole radicals (Li et al., 2017) (Figure 14). H-abstraction reactivities of eight HIRs were investigated for C–H hydroxylation or amidation, and PFBI-OH and BI-OH were proved as the most effective oxidants respectively for tertiary C–H bonds and benzylic C–H bonds. Distinct from the typical radical chain mechanism, the authors proposed a new ionic pathway (Figure 14C) involving nucleophilic trapping of a carbocation intermediate with H_2_O or nitrile cosolvent.

**Figure 15:**
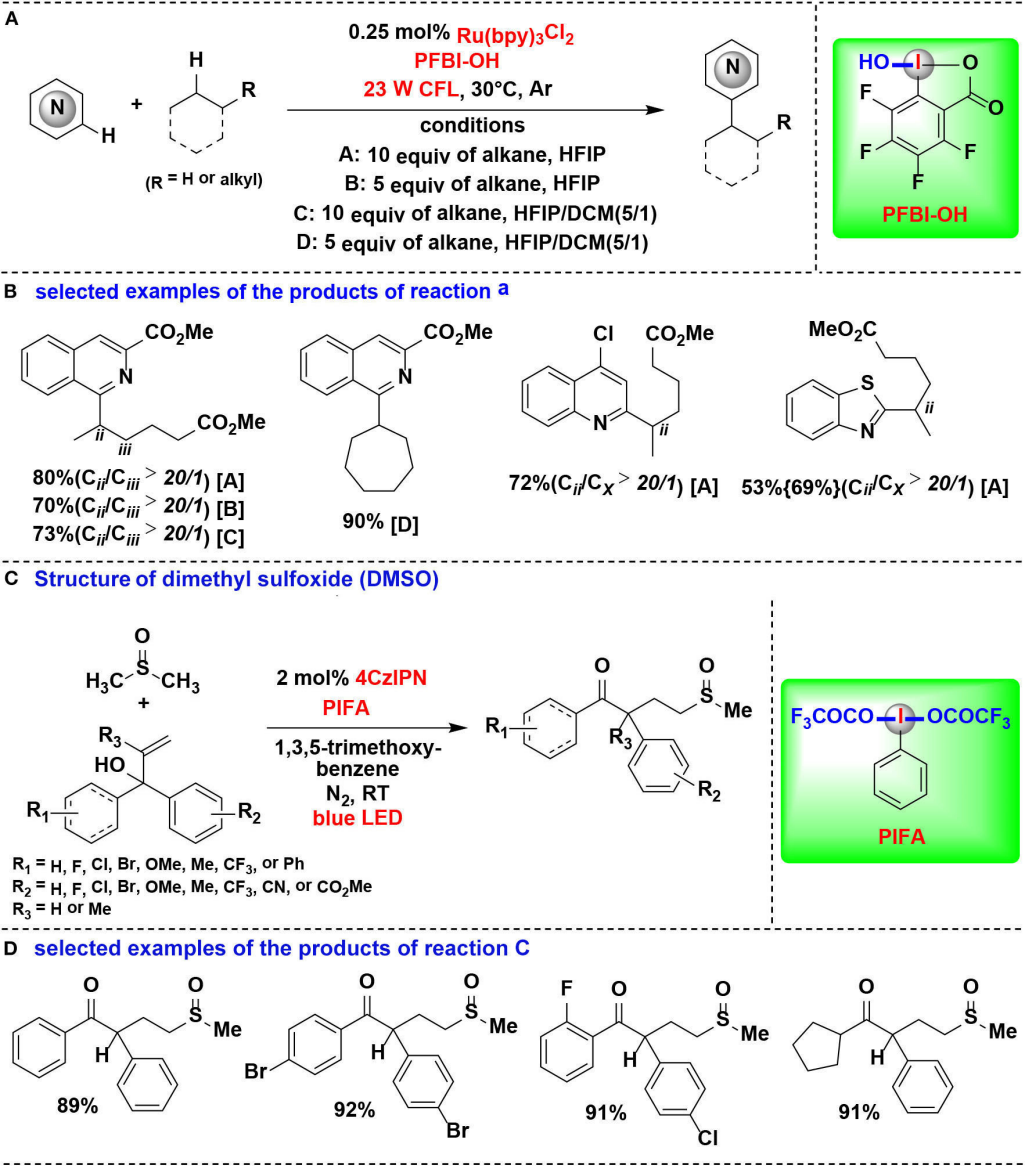
HIR-mediated activation of alkyl C-H bonds under photoredox catalysis. **(A)** Activation of C–H bonds for Minisci-type alkylation of reaction **(A)**. **(B)** Selected examples of the products. **(C)** Activation of C–H bond in DMSO for 1,2-alkylarylation. **(D)** Selected examples of the products of reaction **(C)**.

In an effort focused on extending this methodology, the same authors applied their PFBI-OH/photoredox system to functionalize the challenging methylene C-H bonds, and a range of alkyl-substituted *N*-heteroarenes were efficient and chemoselectively constructed through Minisci-type alkylation reaction of *N*-heteroarenes with alkanes (Figures 15A,B) (Li et al., 2018). The use of PFBI-OH was crucial to elicit both high reactivity and unique steric sensitivity for C-H abstraction of alkanes. The PFBI· radical, which generated by homolytic cleavage of I-OH bond under compact fluorescent lamp (CFL) irradiation, can smoothly cleave stronger 2°C-H bonds even in the presence of weaker 3°C-H bonds.

Cai and coworkers developed a visible-light-promoted C-H functionalization strategy to prepare α-aryl-γ-methylsulfinyl ketones (Figures 15C,D) (Lu et al., 2018). In this process, alkyl C(sp^3^)–H bond of dimethyl sulfoxide (DMSO) can be cleaved by a new HIR to yield α-sulfinyl radical, which subsequent undergoes radical addition with allylic alcohol, followed by 1,2-aryl migration to give the desired sulfoxide derivatives. The new HIR was *in situ* generated from the reaction of PIFA and 1,3,5-trimethoxybenzene.

## Summary and Outlook

As shown herein, the synergistic combination of photoredox catalysis with HIRs has achieved numerous notable organic transformations. These reactions illustrated that hypervalent iodine chemistry can significantly benefit from the merger with photoredox catalysis systems. The ability to access highly reactive radical intermediates under very mild and environmentally benign conditions make these methodologies quite attractive.

Despite the significant progress made, there remain many opportunities for further exploration in the field of photoredox catalysis/HIR system. Firstly, there are a wide variety of HIRs yet to be engaged in photoredox-catalytic reactions. Moreover, from the perspective of green and sustainable chemistry, additional development of low-cost, non-toxic, and environmentally benign organic-dyes as a replacement of metal photoredox catalysts is highly desirable. Additionally, the discovery of stereoselective asymmetric reactions using chiral HIRs under photoredox-catalyzed conditions may potentially be a promising direction for future research. Finally, more in-depth mechanistic studies are highly warranted for fully understanding of the photoredox catalysis/HIR processes. It is highly anticipated that more and more HIRs as reagents or oxidants will continue to be applied in the area of visible-light-induced photoredox catalysis.

## Author Contributions

TY designed this proposal, determined the contents, and revised the manuscript. CC collected the literature data related to this review and wrote the manuscript. XW drew the chemical structures and prepared the figures. All authors contributed to the final version of the manuscript.

## Conflict of Interest

The authors declare that the research was conducted in the absence of any commercial or financial relationships that could be construed as a potential conflict of interest.
